# Rapid, cool sintering of wet processed yttria-stabilized zirconia ceramic electrolyte thin films

**DOI:** 10.1038/s41598-017-12438-9

**Published:** 2017-09-29

**Authors:** Jun-Sik Park, Dug-Joong Kim, Wan-Ho Chung, Yonghyun Lim, Hak-Sung Kim, Young-Beom Kim

**Affiliations:** 1 Department of Mechanical Engineering, Hanyang University, Haengdang-dong, Seongdong-gu, Seoul, 133-791 South Korea; 2 Institute of Nano Science and Technology, Hanyang University, Seoul, 133-791 Korea

## Abstract

Here we report a photonic annealing process for yttria-stabilized zirconia films, which are one of the most well-known solid-state electrolytes for solid oxide fuel cells (SOFCs). Precursor films were coated using a wet-chemical method with a simple metal-organic precursor solution and directly annealed at standard pressure and temperature by two cycles of xenon flash lamp irradiation. The residual organics were almost completely decomposed in the first pre-annealing step, and the fluorite crystalline phases and good ionic conductivity were developed during the second annealing step. These films showed properties comparable to those of thermally annealed films. This process is much faster than conventional annealing processes (e.g. halogen furnaces); a few seconds compared to tens of hours, respectively. The significance of this work includes the treatment of solid-state electrolyte oxides for SOFCs and the demonstration of the feasibility of other oxide components for solid-state energy devices.

## Introduction

With the increasing interest and popularization of renewable energy conversion and storage systems over the past years, much attention has been focused on technologies such as secondary batteries, photovoltaics, electrolysers and fuel cells. It is generally considered that all-solid-state energy conversion and storage devices are the most promising considering the high reliability, efficiency, safety, and flexibility of the systems^1–4^. Solid-state thin film electrodes and electrolytes are essential components of these devices, and hence, they are intensively studied in various fields of materials science and electrochemistry. For example, lithium-sulphur (Li-S) secondary batteries have been continuously studied to replace liquid electrolytes with solid ones^5,6^, and dye-sensitized solar cells use solid state hole transport materials that can be used as a hole conductor instead of liquid electrolytes (that can cause electrode corrosion and electrolyte leakage)^7,8^. These solid-state-electrolyte-based energy devices are attractive as they can potentially compensate for some of the drawbacks of existing technologies.

In the same context, solid oxide fuel cells (SOFCs) are a promising and extensively investigated solid-state energy conversion system as they have environmentally friendly features and excellent energy conversion efficiency^9,10^. In order to attain reasonable power outputs, SOFCs require relatively high operating temperatures (800–1000 °C) to mitigate the low ionic conductivity of the popular yttria-stabilized zirconia (YSZ) electrolyte material. Such a high operating temperature limits commercial development of SOFCs due to the challenges of material compatibility and fabrication costs^11–13^. Recent studies have focused on lowering the operating temperature to 400–600 °C to bypass these challenges; however, this is accompanied by an inevitable decrease in the performance due to the lower ionic conductivity and activation kinetics for charge transfer at these temperatures^14,15^. To compensate for the significant losses at reduced operating temperatures, various nanofabrication methods, including thin film deposition techniques, have been employed to shorten the ionic conduction path^16–18^. Various vacuum and non-vacuum thin film deposition techniques have been investigated to minimize electrode and electrolyte thicknesses for low-temperature SOFC fabrication, and the power output was shown to be satisfactorily high^16,19–23^. However, regardless of the deposition method, a heat treatment process (including post-annealing) is generally required to achieve highly crystalline oxide films with desired properties. As these sintering processes are usually performed at temperatures above 1200 °C in conventional furnaces under controlled environments, the overall fabrication time and cost are high. Furthermore, exposure to a high temperature environment for tens of hours can also limit the choice of suitable materials and cause undesired film-substrate interface reactions. Besides, furnaces can even induce excessive energy use and environmental pollution for the entire process. This limitation in the ceramic processing of thin films is a key obstacle hindering large-scale commercialization of devices incorporating such materials.

For these reasons, many novel techniques had been proposed and investigated for reducing the time and temperature of the ceramic film fabrication process including heat treatment while ensuring acceptable material properties of the thin film oxide. The rapid thermal annealing (RTA) process has been suggested as an alternative to traditional sintering since it can dramatically reduce the ramp time in the annealing process. However, this process still requires long sintering and cooling times, which limits its widespread use^24,25^. In addition, although the microwave irradiation process has been proposed as a promising alternative annealing technique, the high complexity of microwave interactions with materials is a major limitation for the types of materials that can be used^26,27^. In addition, it has been proposed that the excimer laser ablation annealing technique could avoid disadvantages of conventional processes, yet this method has limitations due to a narrow spot size and extremely short wavelength radiation^28,29^. Moreover, controlled environments are necessary for applying these processes.

Here, we propose a novel method for ceramic thin film fabrication and heat treatment using white light flash irradiation in the visible wavelength range. This method could significantly simplify the entire fabrication process, including the post-heat treatment step, while the annealing of the coated oxide film could be performed in a few milliseconds at standard temperature. Here, YSZ thin films were coated using a chemical solution deposition (CSD) method and then annealed using high power, pulsed flashlight rather than employing a conventional furnace process. In this study, thorough investigations were performed on conducting ceramic thin film oxides fabricated using CSD and annealed using pulsed flash irradiation treatment. The irradiated films were compared with conventionally annealed YSZ films and the microstructure, film composition (including organic residue and crystalline phases), and ionic transport properties were analysed.

The results of this study will provide significant impact as it proposes a solution for the fabrication of ceramic films instantly under ambient conditions and therefore, expedite commercialization of solid-state energy conversion and storage devices. It is expected that this research could contribute to the emerging topic of the rapid production of low cost, large area solid state energy devices. Moreover, we believe that the proposed method is highly scalable and not only restricted to solid-state energy devices such as solar cells, secondary batteries, electrolysers, and fuel cells, but could also be applied to other devices employing oxide films. In addition, this method allows a broader selection of materials, including heat-sensitive substrates like flexible polymers, and minimises inter-diffusion effects.

## Results

### Preparation of YSZ thin films

Oxide ion conducting YSZ thin films were prepared using the sol-gel method employing a mixture of metal acetates dissolved in acetic acid (HOAc) and ethylene glycol as complexing and polymerization agent, respectively. This synthesis shares a lot of similarities with the Pechini method that used carboxylic acids and poly-hydroxy alcohols as these corresponding agents^30^. After sufficient heating time, esterification reactions promote the reaction of acid and alcohol and create esters, as shown by the reaction given below:

$${\rm{ROOH}}+{\rm{R}}^{\prime} {\rm{OH}}={\rm{ROOR}}^{\prime} +{{\rm{H}}}_{2}{\rm{O}}$$

The esterification reaction converts the solution into covalent organic networks by making new bonds between the complexing and polymerization agents. These organic compounds can interfere with the transfer of metal cations and maintain the homogeneous distribution of the cations by trapping them. This behaviour can suppress the formation of undesired cation phases, such as precipitates^31^. Here, the metal cations in YSZ precursor solution are also expected to be surrounded by these organic compounds and evenly distributed in the system rather than nucleating and precipitating. However, as HOAc only has one functional group, multiple esterification reactions could be limited and large polymer networks derived from continuous esterification will exist around cations only in insignificant concentrations^32,33^. Considering that no evidence of any precipitations or macro defects was observed for this system, it would be possible to speculate that complexation is not a decisive factor in maintaining the homogeneity of cations and hindering nucleation during solvent evaporation. In addition, considering the poor solubility of the yttrium acetate precursor (below 10 wt.%), the initial cation concentrations used in this study were intentionally low (0.085 M). For this reason, it is reasonable that the kinetics of the cation would be limited during evaporation. In addition, it would take a lot of time to reach supersaturation, so precipitation could be avoided. A similar study was conducted using a modified Pechini process with ethylene glycol and acetic acid for the synthesis of In_2_O_3_-based films^33^. The initial cation concentration was ≤0.125 M to prevent the precipitation of the indium-containing organometallic salt. Similarly, when the initial cation concentration of the YSZ solution was above 0.3 M, a precipitate containing the metal cations started to form and develop macro defects in the resulting film.

YSZ thin film fabrication using the prepared solutions, organic compounds in the deposited films should be removed via a combustion process and the development of the YSZ crystalline phase should occur to achieve the desired properties. These processes gradually and sequentially occur in a conventional furnace during treatment at elevated temperatures for extended periods, as the temperature required for development of the crystalline phase is generally much higher than the calcination temperature. In contrast, for the novel process introduced in this study, the heat treatment was divided into two sequential steps, the pre-annealing and main-annealing steps. By controlling the irradiation energy and processing time, two flash irradiation steps achieved for photonic annealing of the YSZ films within a few seconds. The pre-annealing process was designed mainly to decompose any remaining organics and control the film density, while the main-annealing process ensured the development of a crystalline film.

### Pre-annealing by flash irradiation

**Figure 1:**
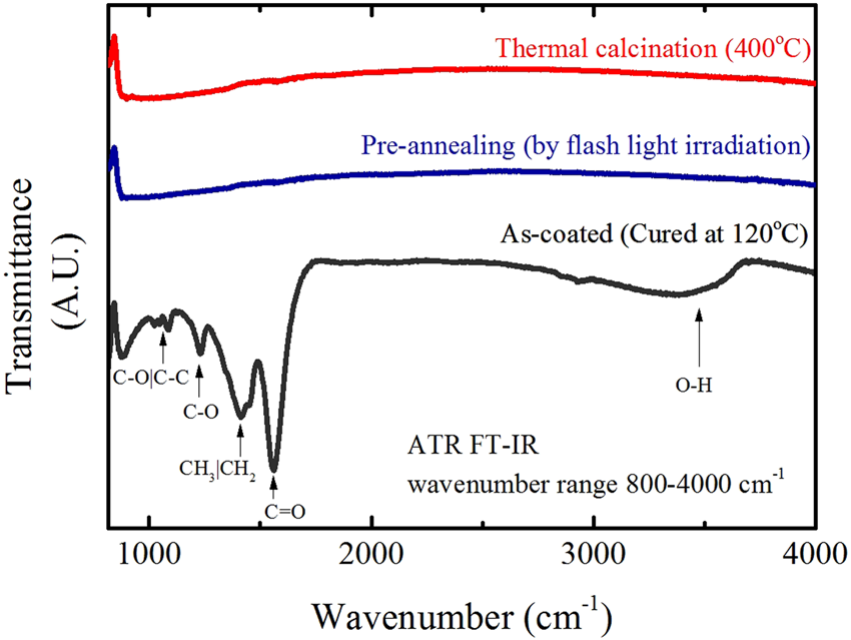
FT-IR analysis data. ATR FT-IR spectra of an as-coated sample (cured at 120 °C), flash irradiated sample (pre-annealing), and thermally calcinated sample (above 400 °C).

Previous studies showed that most of the organic compounds derived from the acetates and alcohols used here could be decomposed above 400 °C, where the film developed amorphous metal oxides^33,34^. The residual organic matter can cause secondary contamination and deteriorate the properties of the film. Hence, FT-IR analyses were performed here to confirm that the processing removed all the organic compounds. As shown by the infrared spectra in Fig. 1, the as-coated sample (cured at 120 °C) clearly shows the existence of organic functional groups, such as carboxylic acids and derivatives at the wavenumber range of 1100–1800 cm^−1^, and the OH functional stretch, expected to have originated from residual water from the solution,was also detected. However, no trace of characteristic organic bands was observed in the samples thermally calcinated at temperatures above 400 °C, implying that the decomposition processes had taken place.

**Figure 2:**
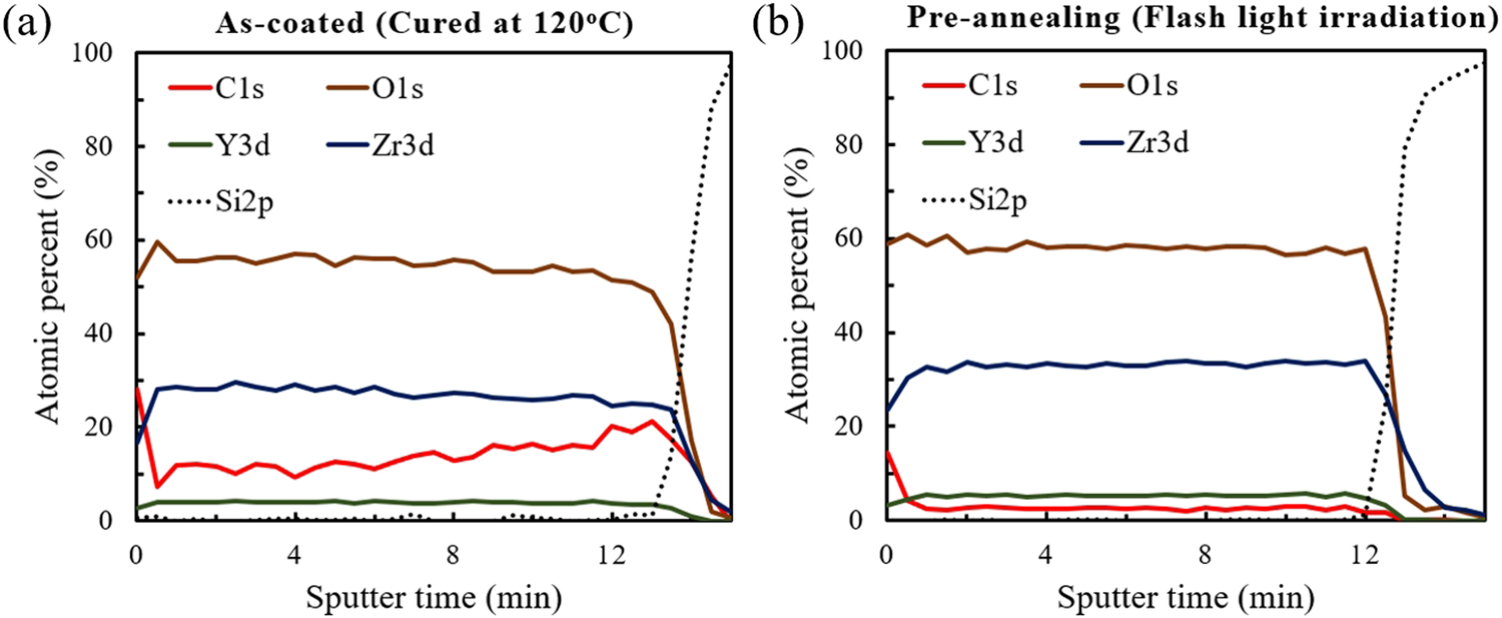
XPS depth profiles (equivalent to the sputtering time) for various elements of interest. (**a**) As-coated (cured at 120 °C) and (**b**) pre-annealed flash irradiated YSZ/Si sample.

Such conventional thermal treatment required several hours to complete the entire process, while the use of flash irradiation sintering took only a few seconds. After the pre-annealing step of the flash irradiation process (300 J/cm^2^ total energy with 30 pulses) the sample showed a similar infrared spectrum to that of the thermally calcinated film, as shown in Fig. 1. Residual organics seemed to be well decomposed and evaporated after the irradiation process, indicating that most of the residual derivatives were able to be decomposed instantly via instant flash irradiation. The irradiated light absorbed by the film instantaneously triggers the photo-activation effect and decomposes remnant organics^35^. To confirm the decomposition of organic compounds across the whole film thickness, XPS measurement with depth profiling was conducted as shown in Fig. 2. A steady sputtering rate was applied by a Ag^+^ ion beam current to obtain the XPS depth profiles of as-coated and flash irradiated YSZ films. Except for the results from the top-surface before Ar^+^ etching, the carbon concentration of the flash irradiated sample clearly decreased and showed consistent YSZ composition (about 8.15 mol% of YSZ) over the whole depth compared to the as-coated film, supporting the results of the FT-IR in Fig. 1. Meanwhile, the amount of detected carbon in the as-coated film was relatively low at the surface, which was expected as the organics were partially evaporated during the curing process. According to these results, it was possible to confirm that the photo-activating effect of the flash irradiation is sufficient for the decomposition of remnant organics in films produced by wet-chemical solution-based methods.

### Main-annealing by flash irradiation

Considering the required annealing temperature for the development of the crystalline YSZ phase, the irradiation energy density required for the main-annealing process was much higher than that of the pre-annealing step. The oxide films synthesised here used carboxylic acid and polyhydroxy alcohol with metal-organic salts; similar studies indicated that annealing temperatures above 700 °C were required to produce well-crystallized films with these precursors^36–38^. A much higher output power density per pulse was required for the main-annealing step and the time interval between each pulse needed to be decreased to achieve intense light irradiation. The entire process time for this step was shortened to about 0.1 s and the total irradiation energy density varied from 50 to 80 J/cm^2^.

**Figure 3:**
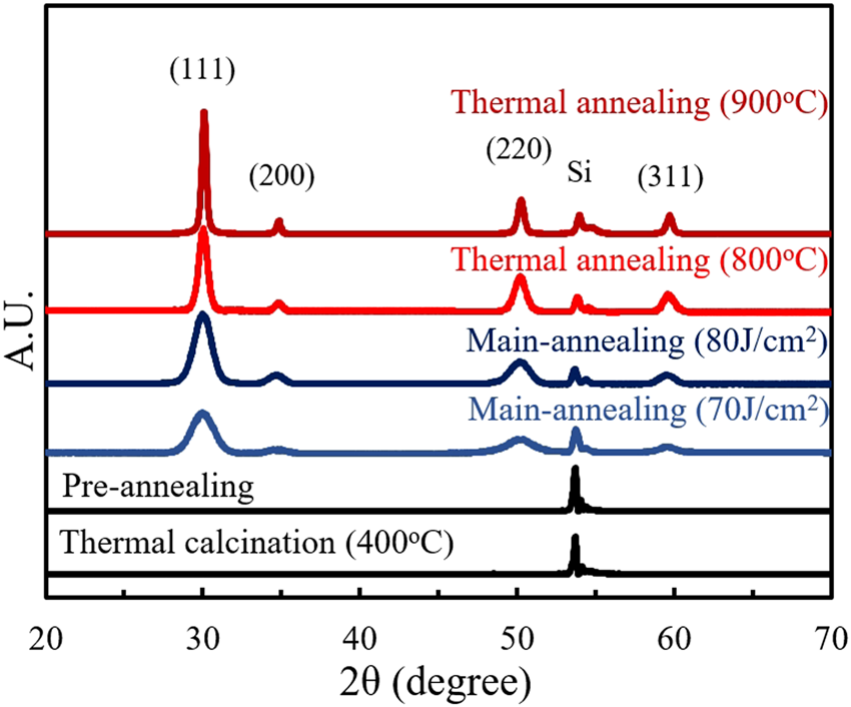
X-ray diffraction patterns of YSZ film coated on silicon wafers; thermally calcinated (400 °C), flash irradiated (pre-annealing step and main-annealing step 70 and 80 J/cm^2^), and thermally annealed (800 and 900 °C). All diffraction patterns were refined using the pseudo-Voigt profile function with asymmetry.

To investigate the development of the crystalline phase of the thermal/photonic annealed YSZ films, x-ray diffraction (XRD) analysis was conducted. The diffraction patterns obtained from conventional thermal annealing and flash irradiation processes are shown in Fig. 3. All XRD pattern refinements were carried out with a peak processing program using a pseudo-Voigt profile with asymmetry. After thermal calcination and pre-annealing, amorphous films were observed, while well-crystallized YSZ films were achieved by flash light irradiation and high temperature thermal annealing; this confirms that the crystallization process occurred in a similar way in both the flash irradiation and thermal heating processes.

The diffraction pattern of the flash irradiated film produced at 80 J/cm^2^ was identical to those of the thermally annealed samples (800 and 900 °C), while samples irradiated below 70 J/cm^2^ did not achieve the crystalline phase and showed an amorphous structure. However, it was observed that this amorphous phase started to turn into the crystalline phase, with significant diffraction from the (111) plane, with irradiation energy densities of 70 J/cm^2^ and higher. In addition, further crystallinity development was observed as the irradiation energy density reached 80 J/cm^2^. This result shows the degree of crystallinity of light irradiated YSZ film can be improved with increasing energy density. However, thermal annealed sample (900 °C) still shows sharper and higher intensity peaks than irradiated ones. According to the full-width-at-half-maximum values derived from de-convoluted diffraction patterns of crystalline films shown in Fig. 3, the grain size of each thermally annealed and light irradiated sample was calculated using the Williamson-Hall equation with a correction for instrumental broadening^39,40^. The estimated average grain size of the sample flash irradiated at 80 J/cm^2^ was about 12 nm, slightly smaller than that of the samples thermally annealed at 800 and 900 °C, estimated to be 18 and 29 nm, respectively.

**Figure 4:**
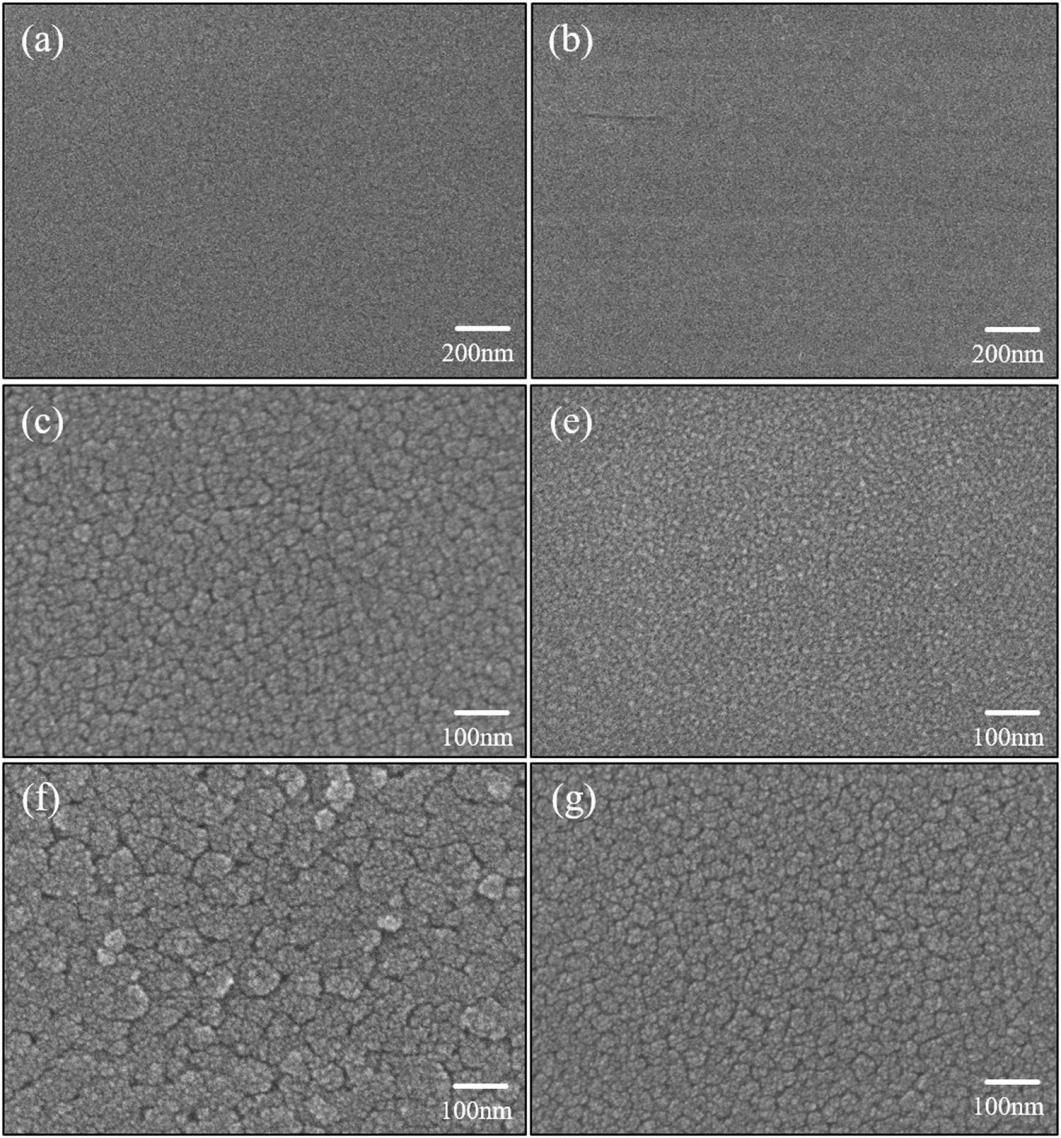
FE-SEM images of the surfaces of YSZ films. Samples (**a**) thermally calcinated at 400 °C, (**b**) flash irradiated (pre-annealing) at (**d**) 70 J/cm^2^ and (**f**) 80 J/cm^2^, and thermally annealed at (**c**) 800 °C and (**e**) 900 °C.

Figure 4 shows surface images of the thermal/photo-annealed YSZ films obtained by field-emission scanning electron microscopy (FE-SEM). The crystalline films showed different surface morphologies to the amorphous films. The crystalline YSZ films showed densely packed grains on the surface, showing that the surface grain size of thermally annealed and flash irradiated samples increased with annealing temperature and irradiation energy, as expected. These results are in good agreement with the XRD results.

**Figure 5:**
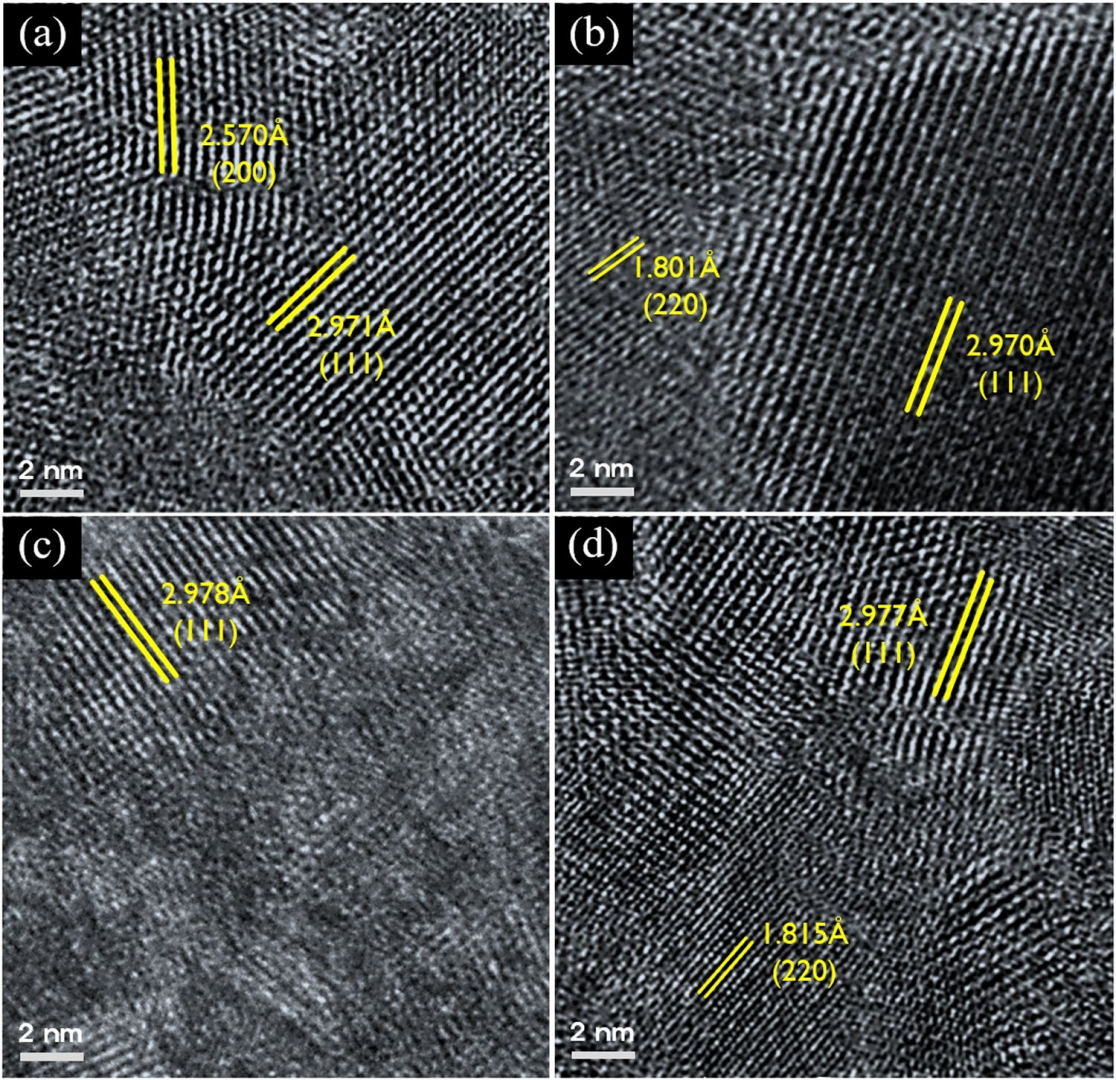
High-resolution bright-field STEM image of YSZ thin films. Samples thermally annealed at (**a**) 800 °C and (**b**) 900 °C, and light irradiated at (**c**) 70 J/cm^2^ and (**d**) 80 J/cm^2^.

To further investigate the structure, scanning transmission electron spectroscopy (STEM) imaging was performed and the results are shown in Fig. 5, where (a),(b), and (d) are the thermally annealed YSZ films at 800 °C and 900 °C, and flash irradiated at 80 J/cm^2^, respectively. Clear crystal planes with certain orientations were confirmed for all of these films, while the STEM image of the film formed at an irradiation energy of 70 J/cm^2^ (Fig. 5(c)) showed that crystalline formation was only just starting to take place.

**Figure 6:**
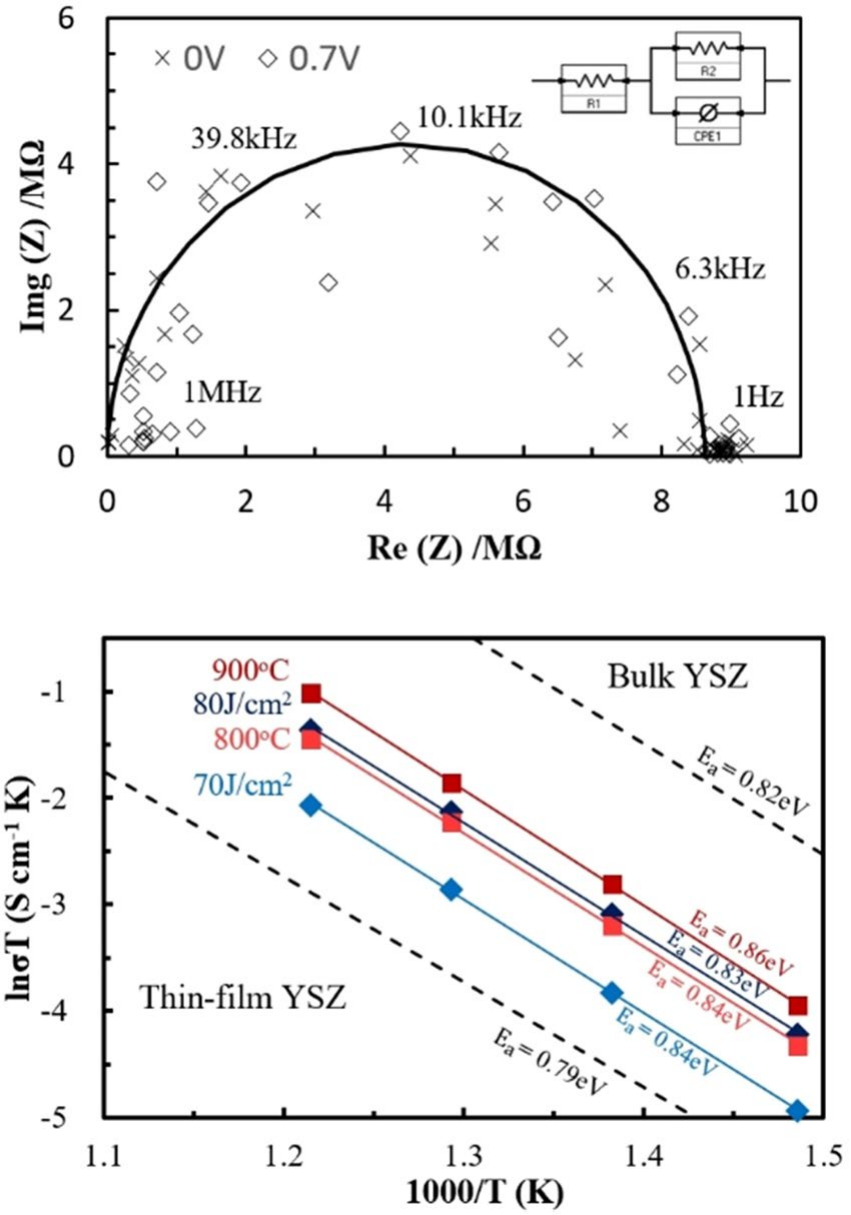
Impedance spectroscopy and conductivity data. (**a**) Nyquist plot of flash irradiated YSZ films deposited on polycrystalline Al_2_O_3_ measured at 550 °C under a DC bias of 0 V and 0.7 V. Inset is the equivalent circuit used to fit the curves. (**b**) Normalized Arrhenius plot of in-plane ionic conductivity (lnσT) of YSZ thin films coated on Al_2_O_3_ (polycrystalline), thermally annealed at 800 and 900 °C, and flash irradiated samples (70 and 80 J/cm^2^). The dotted lines compare bulk and thin film YSZ conductivity values from the literature^45,46^.

The ionic conductivity values of the YSZ thins films were measured using electrochemical impedance spectroscopy (EIS) over the frequency range from 1 MHz to 1 Hz and temperature range of 400–550 °C. Figure 6(a) shows a representative Nyquist plot of in-plane impedance measurement data of a flash irradiated YSZ film deposited on polycrystalline Al_2_O_3_ with applied bias voltages of 0 V and 0.7 V. The impedance results were fitted using an electrical equivalent circuit model (as shown in the inset). The measured EIS semicircle did not vary with applied voltage, confirming that it contributes to ionic transport inside the electrolyte^41–44^. On account of relatively thin thickness of samples (~150 nm) and large distances of Pt electrode stripes (0.25 nm), the resistances were definitely high and consequently cause noises in spectra as shown at Fig. 6(a). Figure 6(b) shows Arrhenius plots of the in-plane ionic conductivity of annealed films extracted from the measured Nyquist plots and compared with reference YSZ conductivity values. The dotted line indicate reference data for bulk and thin film YSZ conductivities from the literature^45,46^. As expected, the ionic conductivity values increased with increasing thermal annealing temperature and flash irradiation energy. Although the film thermally annealed at 900 °C showed higher ionic conductivity than the others, the sample irradiated at 80 J/cm^2^ has a slightly lower ionic conductivity. Considering the difference in the heat treatment time between those two samples, the conductivity value of the flash irradiated sample is remarkable. This difference in conductivity values of the samples thermally annealed at 900 °C and light irradiated at 80 J/cm^2^ is attributed to the better crystallinity of the former (as verified by the sharper XRD peaks in Fig. 3). Other possible explanations could be related to grain boundaries and dopant distribution effects. As mentioned in previous works, it is well known that the resistivity of grain boundaries is generally several orders higher than that of the bulk^47–50^. Grain boundaries react as barriers that interfere with ionic transport and the conductivity can be significantly influenced by their composition and structure. The distribution of dopants, which could be induced by segregation effects, also affects the ionic conductivity behaviour^51,52^. Meanwhile, the ionic conductivities of the thermally and photonic-annealed YSZ films existed in the range between the reference thin film and bulk YSZ, and had similar activation energies (E_a_) to these films, as determined by the slopes of the data sets shown in Fig. 6(b).

These results have shown that YSZ electrolyte films instantly irradiated at ambient temperature and pressure can achieve comparable conductivity behaviour to conventionally annealed samples. It is expected that further improvements may be observed with increasing irradiation energy. The significance of this study lies in the demonstration of a solid oxide electrolyte fabricated by a flash irradiation method; it is expected that this technique could be extended to the fabrication of other solid-state electrolyte devices and will provide a new direction to accelerate commercialization of such devices.

## Discussion

In this study, we demonstrated an extremely simple and effective method for producing solid oxide electrolyte films via the photonic annealing effect during flash irradiation. A wet chemical method using metal-organic precursors and organic solvents was used to produce the precursor film and organics remaining after drying were easily decomposed by a pre-annealing flash irradiation step. By applying higher irradiation power densities, the photo-annealing effect induced crystallization of coated film and hence, good ionic conductivity. The properties of the resulting YSZ films were comparable to those of conventional thermally annealed films; however, the required processing time was dramatically decreased to seconds under ambient conditions at room temperature. This novel approach for sequential treatments of the coated oxide films can offer enormous advantages over conventional thermal annealing methods. It is expected to be applied not only on solid-state electrolyte oxide films for fuel cells, but also other applications of solid-state energy devices such as dye-sensitized solar cells and secondary batteries.

## Methods

### YSZ solution synthesis and film deposition

To obtain 8 mol.% YSZ films, an 84:16% Zr:Y solution was prepared by combining zirconium acetate (Sigma-Aldrich, U.S.A) and yttrium acetate (Aldrich) as precursors. Acetic acid (Fluka, U.S.A) diluted in distilled water and ethylene glycol (99.5% Duksan, South Korea) were used as complexing and polymerization agents, respectively. To get rid of excessive water, the precursor solution was constantly stirred on a hotplate for about 40 hours. The concentration and viscosity of the solution was precisely controlled by adding methanol as a wetting agent. Filtration of the solution was performed using a 200-nm nylon mesh filter to remove undesired precipitates prior to spin coating of the film onto polycrystalline Al_2_O_3_ substrates (MTI Corporation, U.S.A) and silicon (100) wafers and spin coated with thickness of approximately 150 nm. All coated samples were directly dried and cured in 120 °C for 30 minutes. Control samples were heat treated at 800–900 °C for 2 hours in a conventional furnace.

### Flash irradiation annealing

The flash irradiated samples were prepared at room temperature and standard pressure.

**Figure 7:**
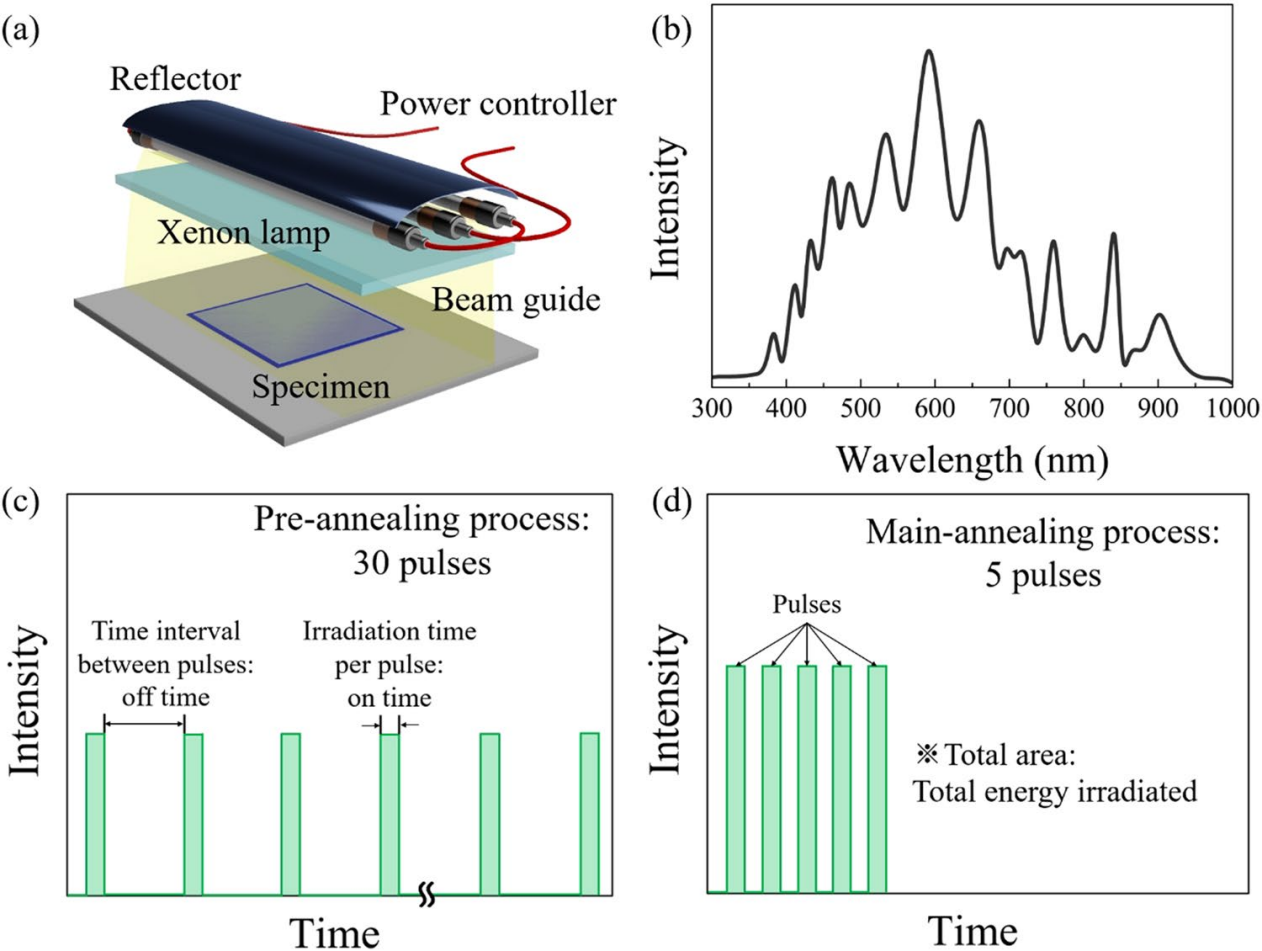
Description of flash irradiation equipment and process. (**a**) Schematic illustration of the flash irradiation equipment. (**b**) Spectrum of the flash irradiation pulse from the xenon lamp. Pulse characteristics from the xenon lamp for the (**c**) pre-annealing step and (**d**) main-annealing step.

Figure 7(a) shows a schematic diagram of the in-house built flash irradiation system. This system is composed of a xenon flash lamp (PerkinElmer Corp., U.K.), aluminium reflector, beam guide, power supply, and controller. The spectrum of the xenon lamp had a broad wavelength range (380–980 nm, as shown at Fig. 7 (b)) which originated from the arc plasma phenomenon^53^. The irradiation conditions, such as irradiation energy density per pulse (J/cm^2^), irradiation time per pulse (on time; ms), interval time between pulses (off time; ms), and number of pulses were varied to optimise the photonic annealing process. In the present study, the flash irradiation process was divided into two steps; pre-annealing and a main-annealing step to produce crystallized YSZ films. The pre-annealing step involved irradiation in 30 rectangular pulses (on time: 10 ms, off time: 100 ms) and a main-annealing step with 5 rectangular pulses (on time: 10 ms, off time: 10 ms) as defined by Fig. 7(c) and (d), respectively. The irradiation energy density per pulse was varied in the main-annealing step (and measured as a total irradiated energy density with a power meter (Nova II, People Laser Tech Inc., South Korea), where all values were higher than those of the pre-annealing step.

### Characterization

In order to characterise the composition of the deposited films and the atomic depth profile, X-ray photoelectron spectroscopy (XPS; PHI 5000 VersaProbe) with a hemispherical analyser and a monochromated Al Kα radiation source [1486.6 eV] (ULVAC PHI Inc., Japan) at power of 24.5 W with background pressure of 2.0 × 10^−7^ Pa. Ar^+^ ion beam sputtering (incident energy of 1 KeV) was used. Depth profiling was conducted by sequential sputtering of the specimens (rastered area: 2 mm$\,\times \,$2 mm) and XPS analysis was performed after each sputtering cycle with spotted area of 0.1 mm$\,\times \,$0.1 mm. Fourier transform infrared spectroscopy (FT-IR: Nicolet iS50, Thermo Fisher Scientific Inc., U.S.A) with Attenuated total reflection (ATR, wavenumber range: 800–4000 cm^−1^) was also performed to analyse the remaining organic materials in the film.

Field emission scanning electron microscopy (FE-SEM: JSM-6701F, JEOL Ltd., Japan) was employed to observe the surface morphology, microstructures, and thickness of the fabricated YSZ films fabricated under different conditions. To confirm the crystalline structures and crystallinity development of the films, X-ray diffractometry (XRD: DMAX-2500/PC, CuKα radiation [λ = 1.54 Å] with 2${\rm{\theta }}$ scan range of 20–70°, Rigaku Co., Japan) was performed. All diffraction patterns were refined using peak processing software (Jade 5.0, Material Data Inc., USA)^54^.

The in-plane ionic conductivity of the films was measured using an in-house built tungsten heating stage with a temperature controller unit under ambient air. Patterned Pt electrode stripes were sputtered on YSZ samples to measure an in-plane resistance with two-probe method. Electrochemical impedance spectroscopy (EIS: Gamry Potentiostat FAS2, Gamry instruments Inc., U.S.A) was performed at fixed voltage conditions of 0 V and 0.7 V, and impedance spectra were obtained through input current with a frequency range of 1 MHz to 1 Hz. The normalized ionic conductivity of each sample was derived from Nyquist plots curve fitted using commercial Gamry Echem Analyst software (ver. 6.25, Gamry Instruments, Inc. U.S.A), displayed as an Arrhenius plot. For further investigation of the crystalline development of photonically annealed YSZ films, Cs-corrected transmission electron microscopy (Cs-TEM: JEM-ARM200F, JEOL, Japan) was performed at an accelerating voltage of 200 kV. The specimens were prepared by ion milling using a Gatan precision ion polishing system (PIPS 691, Gatan Inc., USA) and focused ion beam (FIB) milling was carried out to prepare cross-sections of the YSZ samples.
